# Susceptibility to Aneuploidy in Young Mothers of Down Syndrome Children

**DOI:** 10.1100/tsw.2009.122

**Published:** 2009-10-02

**Authors:** Lucia Migliore, Francesca Migheli, Fabio Coppedè

**Affiliations:** ^1^ Department of Human and Environmental Sciences, University of Pisa, Italy; ^2^ Department of Neuroscience, University of Pisa, Italy

**Keywords:** Down syndrome (DS), mothers of Down syndrome children, trisomy 21, folate metabolism, epigenetics, Alzheimer’s disease (AD), risk factors

## Abstract

We recently observed an increased frequency of binucleated micronucleated lymphocytes in women who had a Down syndrome (DS) child before 35 years of age and the fluorescence *in situ* hybridization analysis revealed that micronuclei were mainly originating from chromosomal malsegregation events, including chromosome 21 malsegregation. That study indicated that women who have a DS child at a young age might have a genetic predisposition to chromosome malsegregation in both somatic and germ line cells. Further studies from our group confirmed increased chromosome damage in blood cells of women who had a DS child at a young age and pointed to a possible role for polymorphisms in folate-metabolizing genes in affecting both chromosome damage and DS risk. In the present article, we review the most recent findings on mechanisms and risk factors for chromosome 21 nondisjunction that lead to DS. Multiple risk factors are likely involved in chromosome nondisjunction; they act at different times in the meiotic process and can be of genetic or environmental (epigenetic) origin. We also discuss the increased risk of developing Alzheimer's disease (AD) later in life that was observed in women who had a DS child at a young age. Studies performed in the last years that have shown that the brain is, in fact, a complex genetic mosaic of aneuploid and euploid cells support the unified hypothesis trying to relate DS, trisomy 21, and AD.

## INTRODUCTION

Down syndrome (DS) or trisomy 21 (MIM 190685) is a genetic disease resulting from the presence and the expression of three copies of genes located on chromosome 21. Since the discovery of Lejeune et al.[1], the phenotype of DS has been associated with trisomy for chromosome 21 (or, in some cases, of a large portion of 21q denoted as the DS critical region).

Primary trisomy 21 leading to DS is caused by the failure of a normal chromosome 21 segregation during meiosis (meiotic nondisjunction) and accounts for 92% of the total of DS cases[2]. In 95% of the cases, primary trisomy 21 is of maternal origin, with the nondisjunction event occurring primarily during meiosis I (MI) in the maturing oocyte, before conception. In contrast, paternal nondisjunction of chromosome 21 during spermatogenesis only accounts for 5% of the cases[3]. Only 5% of the total of DS cases is due to Robertsonian translocations of 21q in one of the parents, the most common being t(14q;21q), resulting in 21q trisomy in the offspring[4]. Furthermore, there is indication that t(14q;21q) occurs primarily during oogenesis[5]. The remaining 3% of DS is due to mosaicism, resulting from errors occurring during the first divisions of the zygote[3]. Individuals with primary trisomy 21 possess three copies of the entire chromosome 21, cases due to Robertsonian translocations have a partial (21q) trisomy, whereas mosaics have both normal and trisomic cells, and the DS phenotype varies as a function of the percentage of trisomic cells present in different tissues[6].

The molecular mechanisms underlying meiotic nondisjunction leading to trisomy 21 are still poorly understood and the major risk factor for trisomy 21 is advanced maternal age at conception[3], while paternal age seems to be insignificant in the etiology of DS[7]. After age 35, the risk of bearing a child with DS increases substantially with increasing maternal age; however, most mothers of DS individuals (MDS) are 35 years or younger, suggesting a genetic susceptibility to early nondisjunction for chromosome 21 in such women[8].

## THE ORIGIN OF MATERNAL CHROMOSOME 21 NONDISJUNCTION

Given the strong correlation between advanced maternal age at conception and increased risk for having a DS child, it was believed that chromosome 21 nondisjunction could be the result of the age-related accumulation of errors that makes the female meiotic machinery less efficient and more error prone. However, despite evidence that advanced maternal age at conception is the major risk factor for trisomy 21, little is still known about the molecular mechanisms responsible for chromosome 21 nondisjunction.

Several studies have related altered levels and positioning of meiotic recombinational events to human nondisjunction. Particularly, studies of trisomy 21 have shown that altered levels or absence of recombination are associated with maternal nondisjunction occurring at both meiosis I (MI) and meiosis II (MII). It was observed that nearly one-half of MI errors are caused by the absence of chromosome 21 recombination during maternal meiosis, whereas the majority of the remaining MI errors had recombination events that clustered at the telomere. In contrast, nondisjunction events due to errors at maternal MII were associated with recombination that clustered at the pericentromeric region of the chromosome, suggesting that all nondisjunction events may be initiated during MI and simply resolved at either of the two meiotic stages[9,10].

Subsequent studies have been performed to search for a possible association between maternal age and altered chromosome 21 recombination patterns. A study of trisomy 21 cases of maternal MI origin, grouped by maternal age, indicated that, effectively in aged women, chromosome 21 nondisjunction could be caused by the age-related accumulation of errors leading to a less-efficient meiotic machinery more prone to chromosomal malsegregation. On the contrary, the absence or the altered placement of recombination during MI is likely to be the cause of chromosome 21 nondisjunction in young women[11]. Further confirmation comes from the observation that, for maternal MI errors, a single telomeric exchange imposes the same risk for nondisjunction, irrespective of the age of the oocyte[12]. On the contrary, the examination of MII errors indicates that the presence of a single exchange within the pericentromeric region of 21q interacts with maternal age-related risk factors[12]. A recent large case-control study provided the following findings: (a) advanced maternal age was significantly associated with both MI and MII errors, and (b) the ratio of MI to MII errors differed by maternal age. The ratio was lower among women <19 years of age and those ≥40 years and higher in the middle-age group[7].

A recent hypothesis suggested that maternal trisomy 21 ovarian mosaicism might be another mechanism leading to DS. The authors analyzed premeiotic ovarian cells obtained from eight phenotypically normal female fetuses, observing trisomy 21 mosaicism in all individuals. Therefore, the authors suggested that disomy 21 in eggs could originate from altered pairing and nondisjunction of chromosome 21 during the meiosis of trisomic ovarian cells, indicating that such a phenomenon could also explain the observed altered recombination patterns of chromosome 21 during meiosis[13].

## FOLATE METABOLISM AND DOWN SYNDROME RISK

Folate metabolism is required for the synthesis of the major DNA-methylating agent S-adenosylmethionine (SAM) (Fig. 1). It is now known that in mice, maternal methyl donor supplementation during gestation can alter the offspring phenotype by methylating specific CpG islands[14]. However, 10 years ago, it was suggested that impaired folate metabolism, resulting from the presence of a functional polymorphism of the methylenetetrahydrofolate reductase gene (*MTHFR*), might be a maternal risk factor for having a DS infant[15]. The hypothesis was that a stable centromeric DNA chromatin might depend on the epigenetic inheritance of specific centromeric methylation patterns and on the binding of specific methyl-sensitive proteins in order to maintain the higher-order DNA architecture necessary for kinetochore assembly. Therefore, it was suggested that pericentromeric hypomethylation, resulting from inadequate folate intake and/or impaired folate metabolism, could impair the formation of the kinetochore, resulting in chromosomal nondisjunction[15].

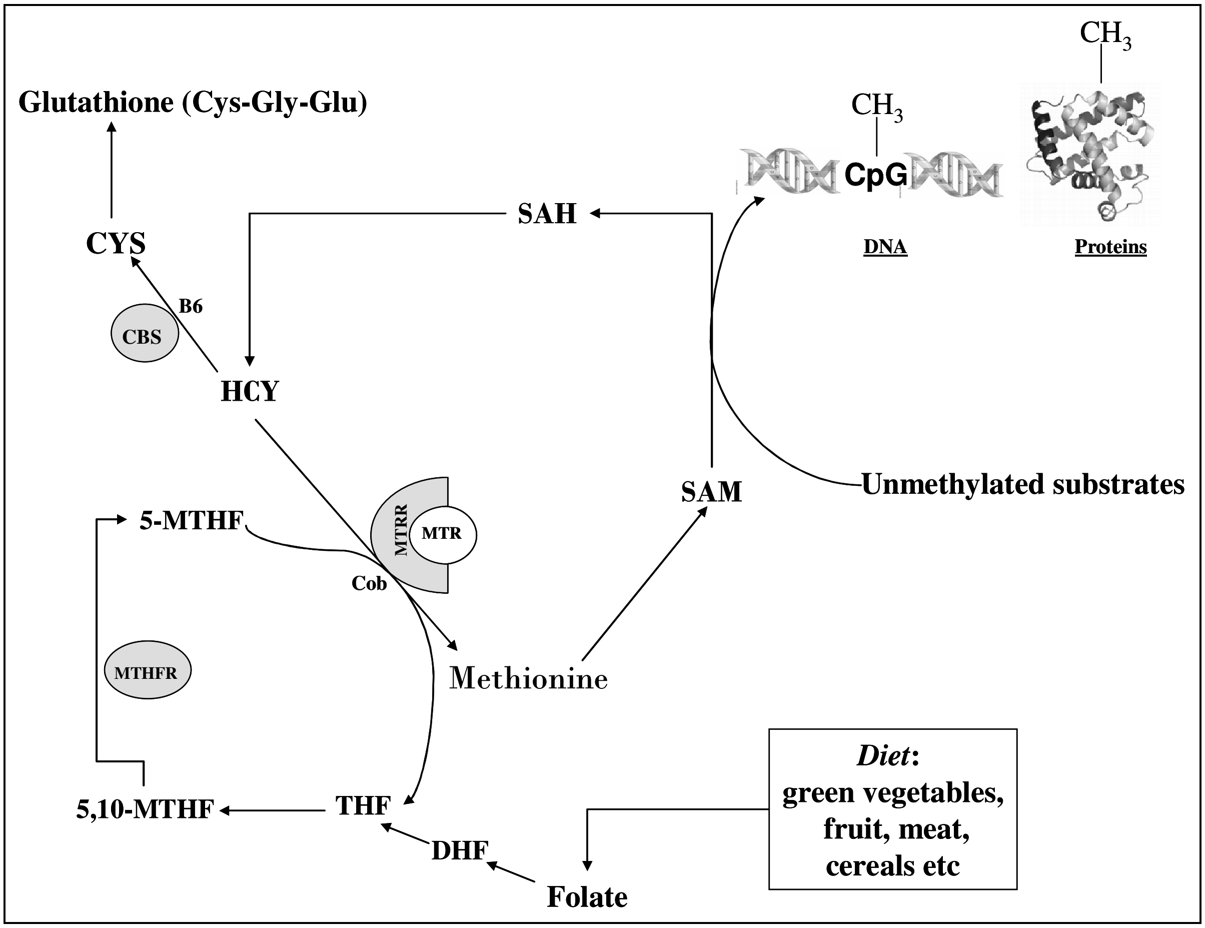

**FIGURE 1**. Folate metabolic pathway. B6, vitamin B6; CBS, cystathionine-β-synthase; Cob, vitamin B12; CYS, cysteine; DHF, dihydrofolate; Glu, glutamic acid; Gly, glycine; HCY, homocysteine; 5-MTHF, 5-methyltetrahydrofolate; 5,10-MTHF, 5,10-methyltetrahydrofolate; MTHFR, methylenetetrahydrofolate reductase; MTR, methionine syntase; MTRR, methionine syntase reductase; SAH, S-adenosylhomocysteine; SAM, S-adenosylmethionine; THF, tetrahydrofolate.

However, as discussed in a previous paragraph, recent evidence suggests that the absence or the altered placement of chromosome 21 recombination during maternal meiosis is responsible for nondisjunction. Therefore, the question is whether or not there could be a link between folate metabolism and chromosomal recombination. Several studies provided evidence that DNA methylation at CpG sites is a mechanism that serves to suppress recombination, providing a direct link between recombination, epigenetic processes, and folate metabolism[16,17]. Particularly, it is clear that crossovers are not randomly distributed, but clustered at specific regions. It has been proposed that DNA methylation might prevent crossover formation, therefore regulating chromosome recombination[16].

Paternal nondisjunction of chromosome 21 during spermatogenesis is the cause of less than 5% of the total of DS cases. Recent studies linked a reduced dietary intake of folate with increased frequency of aneuploidy in spermatozoa, including chromosome 21 disomy[18]. Moreover, there is indication that nonobstructive infertility in males is caused by the absence or the altered position of chromosomal recombination during meiosis[19], and associated with polymorphisms in folate-metabolizing genes[20].

Studies performed on cultured human cells demonstrated that folate deprivation is able to induce chromosome 21 aneuploidy[21], thus linking aberrant folate metabolism to the hypothesis that some DS cases could originate from maternal mosaicism.

Several studies were performed in the last years that were aimed at clarifying the possible role of impaired folate metabolism to DS risk; however, the question is still debated and none of the studied polymorphisms of metabolic genes can be considered undoubtedly as an independent DS risk factor, whereas there is increasing evidence that combinations of two or more of them might affect the risk for a DS pregnancy[22,23,24,25,26,27]. Complex interactions between genetic (metabolic polymorphisms) and environmental factors (folates and related nutrients) have also been suggested[28]. Particularly, most of chromosome 21 nondisjunction events initiate at maternal MI during maternal embryogenesis in the grandmother's body, pointing to a possible role for the maternal grandmother's diet in DS risk[27,29]. Moreover, several of the genes located on chromosome 21 participate in folate metabolism and are overexpressed in DS individuals, possibly resulting in increased folate demand and/or availability in developing DS fetuses, and suggesting that complex interactions between the maternal folate intake during gestation and the trisomic fetal genotype might be relevant for the fetus to develop and survive up to the birth[28].

We obtained similar indications (i.e., gene-gene interactions between *MTHFR* and other genes participating in folate metabolism) when analyzing seven different polymorphisms in folate metabolic genes as possible risk factors for having a DS child at a young (<35 years) maternal age. A first report by us suggested that combinations of *MTHFR* gene polymorphisms with the 80G>A polymorphism in the reduced folate carrier gene (*RFC1*) might impair DS risk[24]. That study is of particular interest since similar or complementary indications came from subsequent independent studies performed by other research groups[26,30]. More recently, we suggested interactions between *MTHFR* variants with other genes of the folate pathway, namely thymidilate synthase (*TYMS*) and methionine synthase (*MTR*), as possible DS risk factors at a young maternal age[27]. This study, recently published, is pending replication in other populations.

Moreover, studies performed in other human aneuploidies than trisomy 21 indicate association of the *MTHFR* 1298A>C polymorphism with Turner syndrome[31] and the *MTHFR* 677C>T polymorphism with trisomy 18[32].

## CYTOGENETIC FINDINGS IN YOUNG MOTHERS OF DOWN SYNDROME CHILDREN

We performed a study aimed at evaluating cytogenetic characteristics, through the micronucleus test, of peripheral blood lymphocytes of a group of women who had a DS child at a young age, in comparison with a control group of mothers who had a healthy child. Moreover, we evaluated the presence of any susceptibility to malsegregation events in a subgroup of mothers by applying the fluorescence *in situ* hybridization (FISH) with molecular probes for chromosomes 13 and 21. A significant increased frequency of binucleated cells with micronuclei was observed. By using probes for chromosomes 13 and 21, the FISH revealed that micronuclei mainly originated from malsegregation events, rather than from chromosome breakage[33]. The study suggested that young MDS might have a generalized tendency to chromosome malsegregation events, not limited to gametes, but detectable even in somatic cells, which could be partially determined by genetic factors.

To further address this question, we subsequently increased the number of MDS under study and searched for a possible correlation between polymorphisms in several folate-related genes and the frequency of binucleated micronucleated lymphocytes (BNML). Those studies confirmed our previous observation of an increased frequency of BNML in MDS respective to controls, and pointed to an association between *MTHFR* gene polymorphisms, namely 677C>T and 1298A>C, and the frequency of BNML in both MDS and controls. However, it also emerged that the *MTHFR* locus alone cannot account for the overall observed increased chromosomal damage in MDS respective to control mothers, suggesting a polygenic contribution in addition to that of still-unclear environmental factors[27,34].

## YOUNG MOTHERS OF DOWN SYNDROME CHILDREN AND ALZHEIMER'S DISEASE RISK

There is a link between DS and Alzheimer's disease (AD). The evidence is that by the fourth decade of life, individuals with DS inevitably develop many of the neuropathological features found in AD and the majority of these individuals develop dementia[35]. The hypothesis is that constitutive trisomy 21 can have a role in brain dysfunction, likely due to the overexpression of genes mapping to chromosome 21, in particular that coding for amyloid precursor protein (APP; senile extracellular plaques are composed of beta-amyloid peptides derived from proteolytic processing of APP).

Among individuals prone to undergo AD are mothers who had a DS child at a young age; an increased frequency of AD (about fivefold) among young MDS is reported[8,36]. A unifying hypothesis trying to relate DS, trisomy 21, and AD was developed in 1991 by Potter[37]. He proposed that trisomy 21 mosaicism at germ cell level or in brain cells could account for the familial aggregation of AD and DS[37].

Studies performed in the last years have shown that the brain is, in fact, a complex genetic mosaic of aneuploid and euploid cells[38]. The possible function of neural aneuploidy and mosaicism could include contributions to cellular diversity, cellular signaling, and diseases of the central nervous system[38]. Aneuploidy in the cerebral cortex of normal and AD brains was recently confirmed by molecular cytogenetic approaches, using DNA probes for different chromosomes, including chromosome 21. The overall proportion of aneuploid cells in the normal brain has been estimated at approximately 10%. Compared with sex- and age-matched controls, the level of stochastic aneuploidy in the AD brain was not found to be significantly increased. However, a dramatic 10-fold increase of chromosome 21–specific aneuploidy was detected in the AD cerebral cortex[39]. Mosaicism for trisomy 21 in brain cells could thus, according to Potter's hypothesis, be critical for AD development; a case of young-onset AD due to occult mosaicism for trisomy 21 (10%) was indeed reported in a patient with no previous diagnosis of DS and only minimal physical manifestations of DS[40]. Somatic mosaicism of cells euploid, but with a mutation in a gene critical for the familial forms of AD (inherited as an autosomal-dominant trait), was reported in a sporadic early-onset patient with AD. He resulted as a somatic mosaic for a mutation in the presenilin-1 gene and the degree of mosaicism was at 8% in peripheral lymphocytes and 14% in the cerebral cortex[41].

Indeed, genomic (chromosomal) instability was shown in other tissues than the brain of AD patients. An increased level of chromosome 21 aneuploidy was observed in lymphocytes, fibroblasts, and buccal mucosa cells of AD patients[42,43,44,45]. To these findings, we can juxtapose our data obtained in lymphocytes of MDS, which were found more prone to nondisjunction events for chromosomes 13 and 21[33]. The hypothesis we suggested in that paper was that those mothers are thus more prone to chromosome malsegregation, and it could be true both for somatic (peripheral blood lymphocytes, brain) as well as for germ cells.

Finally, we would like to mention the fundamental finding that neurons, believed until a decade ago to be in a terminally differentiated postmitotic quiescent state, can re-enter the cell division cycle. Specifically in AD, susceptible neurons exhibit phenotypic changes characteristic of cells re-entering the cell division cycle. Even if it is a relatively rare event, the re-entry into a coordinated cell cycle culminates in nuclear division[46]. It has been demonstrated that various mitogenic signals can cause cell cycle re-entry of neurons in the central nervous system of AD patients, such as loss of synaptic connections and levels of plasma homocysteine (Hcy)[47]. An interesting recent hypothesis predicted that cell cycle re-entry in AD is highly regulated by centromere cohesion dynamics[47]. The sequential separation and segregation of centromeres in the metaphase-anaphase transition is genetically controlled and it has been shown that this sequence of temporal order is altered in AD, i.e., centromeres divide prematurely. This aberrant division is called premature chromosome separation and is seen as a manifestation of genome instability; it has been found in aging, in other chromosome instability syndromes, and *in vitro* following certain chemical treatments, as well as in AD[48,49]. At present there is agreement about the fact that aneuploidy and enhanced neurogenesis are hallmarks of the pathology of AD[50]. The confirmation that neurogenesis occurs in the adult brain and neural stem cells in the adult central nervous system also suggests interesting consequences, such as that the brain may be amenable to repair[51].

In view of the important role of folate metabolism already elucidated above, and considering that AD is characterized by high Hcy and low folate blood levels, meaning that the conversion of Hcy to methionine is altered, as is the production of SAM[52,53], it is likely that an impaired folate metabolism might contribute to the genomic instability observed in AD individuals. A deregulation of a proper chromosome segregation could be the initial event that leads to a clear manifestation of chromosome instability (aneuploidy, binucleation), which may contribute to neuronal degeneration and subsequent cognitive deficits in AD.

## CONCLUDING REMARKS

Overall, the birth of a DS child seems to result from genetic (i.e., polygenic contribution of maternal and fetal folate–metabolizing genes and chromosomal mutations, meiotic chromosome pairing, recombination, or the detection of errors in these processes) and environmental factors (maternal age, maternal and maternal grandmother diet at pregnancy), as well as epigenetic (DNA methylation) and stochastic events (age-related accumulation of damages to biological components participating in chromosomal segregation). Therefore, further studies are required in order to understand the single contribution of each of them. In the light of an interesting point of view that describes human nondisjunction likely as a multifactorial trait[12], even if we are still far from the understanding of the basis of the susceptibility to aneuploidy of MDS, we can outline the scenario shown in Fig. 2.

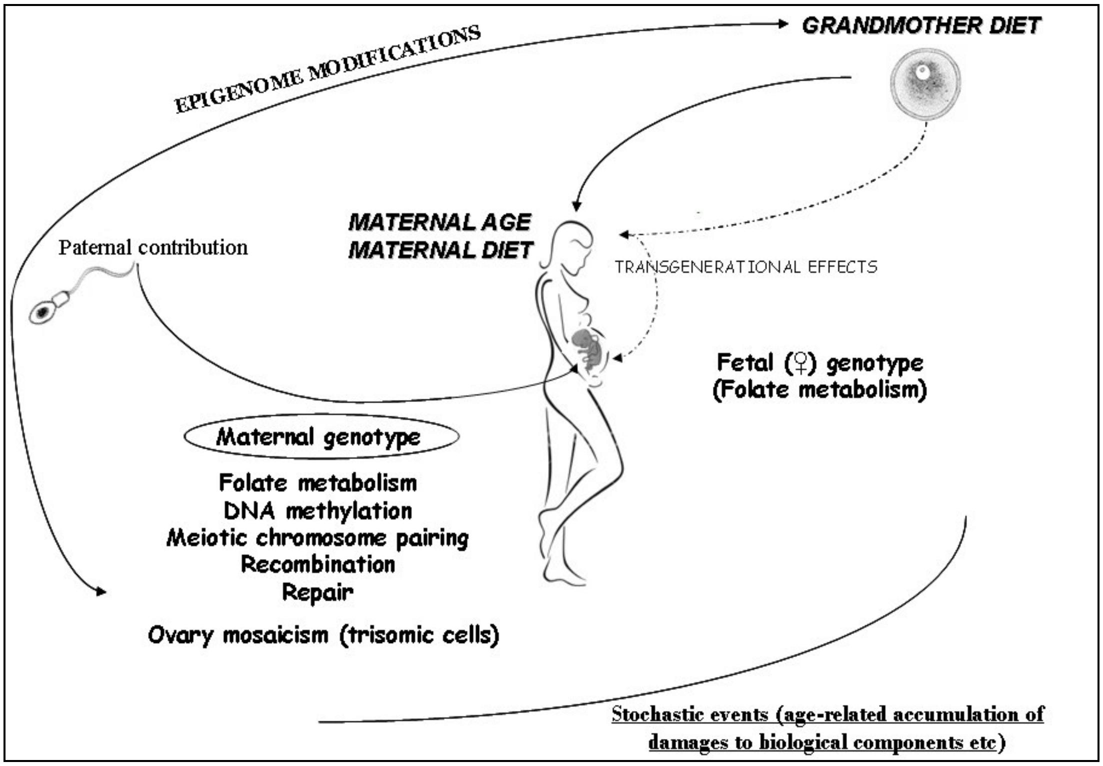

**FIGURE 2.** The human nondisjunction is a complex trait[12] likely due to an interaction between environmental factors and genetic predisposition. In MDS children only few risk factors have been identified so far.
